# Adoption Studies

**Published:** 1995

**Authors:** Remi J. Cadoret

**Affiliations:** Remi J. Cadoret, M.D., is a professor of psychiatry at the University of Iowa, College of Medicine, Iowa City, Iowa

**Keywords:** adoption study, AODR (alcohol and other drug related) problems, hereditary factors, environmental factors, research and evaluation method, behavioral problem, gene

## Abstract

Researchers use adoption studies to determine the contributions of genetic and environmental factors to the development of alcohol problems. These studies generally compare the outcomes of adoptees who have biological parents with alcohol problems and who grow up in various adoptive environments with the outcomes of adoptees without such family backgrounds but raised in similar environments. Using certain statistical approaches, adoption studies also allow for the evaluation of specific gene-environment interactions in determining an outcome such as alcoholism. To obtain data that allow meaningful and generalizable conclusions, however, scientists must select a representative group of study subjects, obtain valid information about these subjects from a wide variety of sources, and consider biases inherent in adoption practices.

Adoption studies are a powerful tool for evaluating the interactions of genetic and environmental factors in eliciting human characteristics, such as intelligence (i.e., IQ), and disorders, such as alcoholism. The relative importance of “nature” (i.e., genetic inheritance) versus “nurture” (i.e., the rearing environment) in human behavior was first debated at the beginning of this century. Simultaneously, some techniques were developed that are still used to study the inheritance of behaviors, including the family study; the twin study (see the article by Prescott and Kendler, pp. 200–205); and statistical methods, such as regression analysis. One pioneer of human genetics, Sir Francis Galton, used these techniques in his studies. Galton concluded from his investigations that “nature prevails enormously over nurture” (Pearson 1914–30). In 1912, one year after Galton’s death, another researcher, L.F. Richardson, proposed to study children who had been separated from their birth parents in order to investigate the inheritance and development of intelligence (Richardson 1912–13).

Concurrent social changes led to greater public acceptance of adoption and also improved researchers’ access to adoptees. For example, foundling societies and orphanages promoted adopting orphans or children born out of wedlock into foster families who were mostly nonrelatives. Adoptive parents usually received little information about the adoptees’ biological parents. The lack of information may have been attributable to the belief at that time in the environment’s overwhelming importance on a child’s development. In addition, having a child out of wedlock was considered shameful, and consequently, confidentiality protected the birth mother. These “closed” adoptions were advantageous for conducting adoption studies because they clearly separated the biological and environmental influences on the adoptee.

In contrast, during the past two decades, a movement has occurred toward more “open” adoptions, in which biological and adoptive parents receive information about each other. Furthermore, this type of adoption may encourage continuing contact of the birth parents with both the adoptee and the adoptive family. In addition, social changes have drastically reduced the number of infant adoptees. For example, most unwed mothers now keep their children rather than give them up for adoption. These developments have increased the practical problems involved in finding and recruiting suitable adoptees for studies.

Between the 1930’s and 1950’s, most adoption studies examined the heritability and effects of environmental influences on IQ. For example, during the 1930’s, Skodak and Skeels (1949) demonstrated increases in IQ in certain environments using an adoption paradigm.^1^ Since the 1960’s, however, adoption studies have been used primarily to demonstrate the importance of genetic factors in psychopathological disorders, such as schizophrenia, alcoholism, or depression (for review, see Cadoret 1986). This article briefly examines some of the principles of adoption studies and the considerations required for their effective evaluation.

## Influences on Adoptees’ Behavior

The strength of the adoption design—separating genetic from environmental influences on a person’s development—results from removing the child (ideally at birth) from the birth parents and their environment into a different environment with biologically unrelated adoptive parents. Thus, adoption studies assess “real-world” influences on the adoptee’s development while allowing for the separation of genetic and environmental factors that are confounded when children are reared to adulthood by their birth parents.

The adoptee’s development and behavioral outcome result from multiple influences exerted by the birth parents and their environment and by the adoptive parents and their environment (for more information on these influences, see sidebar, p. 199). Determining the contributions of these different influences is a multivariate statistical problem. Several statistical techniques, such as multiple regression analysis and log-linear analysis, can address such problems and have been used in evaluating adoption studies. Bohman, Cloninger, and their research group pioneered the use of multivariate approaches for studying the genetics of alcoholism in their analyses of Swedish adoption data (Bohman et al. 1982; Cloninger et al. 1982; Sigvardsson et al. 1982). Using these methods, the investigators assessed the contributions of both genetic and environmental factors on the development of alcoholism in the adoptees.

**Sources of Influences Affecting Adoptee Outcome**

A multitude of influences on the adoptee play a role in determining the adoptee’s development and behavioral outcome. The left side of the diagram (the vertical line represents the separation of biological- and adoptive-family factors) indicates the influences affecting the adoptee during pregnancy, delivery, and the immediate neonatal period, including genetic predispositions inherited from the birth parents (arrow 1) and prenatal and neonatal environmental influences (e.g., maternal alcohol consumption during pregnancy; arrow 3). These genetic and environmental factors also interact with each other, as represented by arrow 5 (e.g., genetically determined antisocial personality disorder or depression in the mother may contribute to her alcohol consumption).

The factors on the right side of the diagram represent the postnatal influences on the adoptee (which, in turn, are influenced by the child) following placement with nonrelatives. Adoptive-parent characteristics are the most important influences affecting the adoptee (arrow 2). The two-headed arrow indicates that the child-parent relationship is an interaction of many factors (e.g., child temperament and parenting skills of adoptive parents). Arrow 4 indicates the correlation between the adoptee and environmental influences. Factors such as friends outside the family influence the adoptee, but the adoptee often simultaneously exerts an influence by seeking out those friends in the first place. Finally, adoptive-parent characteristics and environmental factors also interact with each other (arrow 6). Parent characteristics influence factors such as socioeconomic status. Environmental factors, in turn, can influence parents (e.g., financial stressors may affect parenting behavior by causing depression and irritability).

**Figure:**
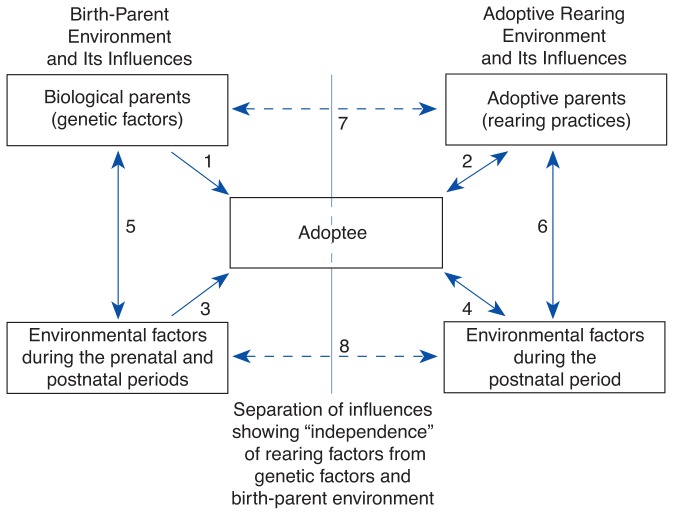
Diagram showing sources of factors that affect adoptee outcome.

In addition, adoptees may be matched to a certain extent to prospective parents based on a variety of factors that can lead to correlations between the biological and the adoptive environments (broken arrows). For example, the educational levels of birth parents and adoptive parents could be used as the basis for matching (broken arrow 7). Similarly, a correlation could exist among environmental factors (e.g., both birth parents and adoptive parents live in rural areas; arrow 8).

—*Remi J. Cadoret*

### Selective Placement and Other Confounding Factors

To allow valid conclusions about the relative influences of genes and environment on adoptee outcome, it is essential that factors originating from the birth parents and their environment are unrelated to, and do not interact with, factors originating from the adoptive environment. This condition could be fulfilled by randomly placing infants in adoptive homes. However, adoption usually is not a random process. Adoption agencies carefully screen adoptive parents, and practical placement decisions frequently result in the selection of older, more stable families; families in higher socioeconomic brackets; and intact, rather than single-parent, families. Conversely, families that give up children for adoption commonly are single-parent, low-income ones.

In addition, adoptees may be matched to prospective adoptive parents depending on a variety of factors. For example, at one time adoptees often were matched with adoptive parents based on physical characteristics, such as hair and eye color. Other, more subtle matchings could depend on psychosocial characteristics. For example, an adoption agency might estimate a child’s “potential” from birth-parent characteristics (e.g., education or socioeconomic level) and place the child according to some expectation of future performance. Finally, racial and ethnic origins also could play a role in placement decisions. These practices, referred to as “selective placement,” could confound the normal contributions of biological and environmental factors. This possibility has led to criticism of adoption studies (Lewontin et al. 1984).

## Design and Evaluation of Adoption Studies

Adoption studies generally can be classified based on whether the adoptees or the birth parents are the probands (i.e., the initial subjects) of the study (Rosenthal 1970). In the adoptees’ study method, researchers identify proband birth parents with a certain characteristic (e.g., alcoholism) and then examine the outcome of these probands’ adopted-away children. A contrasting design is the adoptees’ family method, in which researchers identify proband adoptees with a certain characteristic (e.g., alcoholism or depression) and subsequently examine both the birth and adoptive parents. Both designs have been used to demonstrate the importance of genetic factors in the development of alcoholism. Whether the adoptees’ study method or the adoptees’ family method is used often depends on certain considerations, such as practicality and the ease of recruiting probands and gathering information about them.

Most adoption studies have used a design comparing high-risk probands (i.e., adoptees or birth parents) having certain characteristics (e.g., alcoholism) with a control group of subjects who lack the pathology of the high-risk group and are considered “normal.” In the adoptees’ study design, researchers usually compare the outcome of adoptees with contrasting biological backgrounds (e.g., alcoholic versus nonalcoholic birth parents); further control can be obtained by matching the proband and control birth parents on variables such as socioeconomic level or age. In the adoptees’ family design, the study compares the biological backgrounds of proband adoptees with those of control adoptees, who usually have been selected for normality. In addition, the adoptees may be matched on variables such as age, gender, and socioeconomic level.

**Figure 1:**
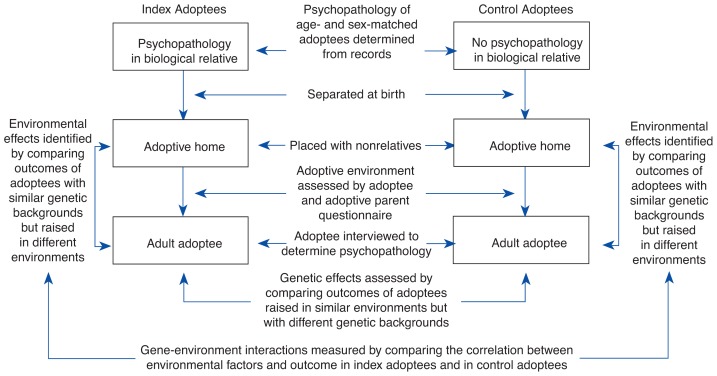
An example of an adoption study using the adoptees’ study method comparing two groups of adoptees: index adoptees and control adoptees.

**Figure 2:**
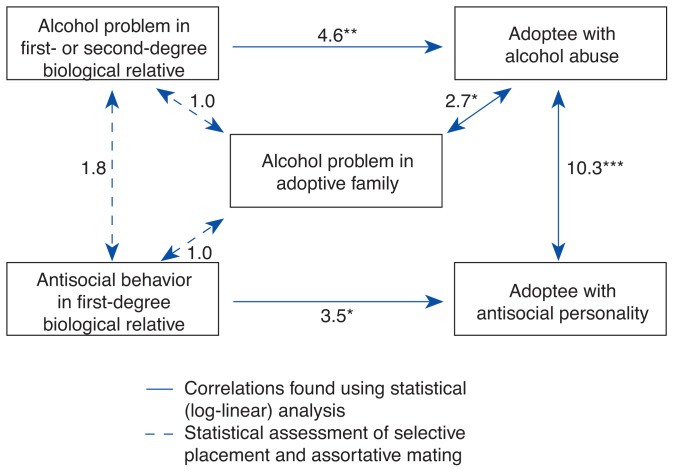
Results of an adoptees’ study method adoption paradigm based on 160 male adoptees and their biological and adoptive families assessed for alcoholism, antisocial personality disorder, and other psychological parameters. The numbers next to the arrows are odds ratios.^1^ (For example, an adoptee with first- or second-degree biological relatives with alcohol problems is 4.6 times more likely to abuse alcohol than an adoptee without such a family background.) ^*^p < 0.05 ^**^p < 0.01 ^***^p < 0.001 ^1^An odds ratio is a measure of association between two variables. SOURCE: Adapted from Cadoret et al. 1987.

A typical adoptees’ study design compares so-called index adoptees—adult adoptees who have backgrounds of psychopathology (e.g., alcoholism) in their biological families—with age- and sex-matched control adoptees who have no family histories of psychopathology. (For a more detailed description of the design of an adoptees’ study paradigm, see figure 1.) An adoption study by Cadoret and colleagues (1987) illustrates how the contributions of several genetic and environmental factors to the development of alcoholism can be determined using this method (figure 2). In the study, 160 male adoptees, their biological relatives, and their adoptive families were analyzed regarding alcohol problems, antisocial behavior, and other psychological variables. The study found that a genetic influence, such as alcohol problems in first-degree (i.e., parents) or second-degree (i.e., grandparents) biological relatives, increased an adoptee’s risk for alcohol problems 4.6-fold. Similarly, an environmental influence, such as alcohol problems in a member of the adoptive family, resulted in a 2.7-fold higher risk for alcohol problems in the adoptee, compared with adoptive families without alcohol problems.

Because the adoption agencies often were aware of both alcoholism and antisocial behavior in the biological parents, these factors could have influenced placement decisions and correlated with the environmental factor of adoptive family alcohol problems. To control for such potential selective placement effects, the correlations between alcohol problems or antisocial behavior in the biological family and alcohol problems in the adoptive family also were assessed in the statistical analysis (figure 2). The study found no evidence of selective placement based on the factors shown: As indicated by the odds ratios^2^ of 1.0, the likelihood of a member of the adoptive family having alcohol problems was the same whether or not biological relatives of the adoptee displayed alcohol problems or antisocial behavior.

### Assortative Mating

Another factor that can affect a child’s development and behavior is assortative mating (i.e., the nonrandom choice of a partner based on personal characteristics). For example, an alcoholic person may be more likely than a nonalcoholic person to have an antisocial or alcoholic partner, possibly because of shared traits or behaviors. The combination of two genetic predispositions may enhance the predisposition of the offspring to develop any psychopathology. Multivariate statistical analyses can help control for the effects of assortative mating if relevant information is available on both birth parents. Similar analyses also can be used to control for the genetic predisposition for two disorders (e.g., alcoholism and antisocial personality disorder) within one person.

### Alternative Evaluation Methods

Simpler statistical analyses also have been used to evaluate the results of adoption studies. For example, when the assessment of genetic influences is the main objective, a common strategy is to demonstrate that the environmental influences are the same for adoptees from high-risk backgrounds (i.e., with alcoholic biological family members) and low-risk backgrounds (i.e., without alcoholic biological family members). Comparable environmental factors for both groups would indicate that no selective placement occurred that could confound the study results. Using this method, Goodwin and colleagues (1973) demonstrated the importance of a genetic predisposition to the development of alcoholism. However, although environmental influences may be similar when averaged over high- or low-risk adoptee groups, considerable environmental variability still exists among the members of each adoptee group that could affect the outcome of individual adoptees and which should be assessed by multivariate statistical approaches.

### Gene-Environment Interactions

**Figure 3:**
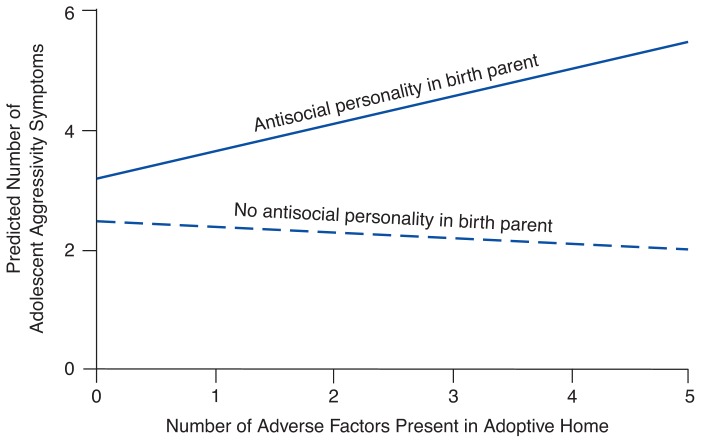
Correlation between antisocial personality disorder in a birth parent, adverse environmental factors in the adoptive home (i.e., marital, legal, psychiatric, or substance abuse problems in the adoptive parents), and adolescent aggressivity in the adoptee. If a birth parent has an antisocial personality, a positive correlation exists between adverse environmental factors and aggressivity symptoms in the adoptee. This correlation is significantly different when none of the birth parents has an antisocial personality. SOURCE: Adapted from Cadoret et al. 1995.

In determining the contributions of genetic factors to an outcome such as alcoholism, it is important to know whether a genetic factor exerts its effect only in the presence of a specific environmental condition or does so independently of environment. The adoption paradigm is a powerful tool for evaluating the interaction of specific genetic factors with specific environmental factors that affect adoptee outcome (DeFries and Plomin 1978). For example, researchers and clinicians have long recognized that both conduct disorder and aggressivity predispose an affected person to alcohol and other drug abuse (see figure 2). Adoption studies also have demonstrated that antisocial personality disorder in birth parents predisposes adopted-away offspring to both conduct disorder (Cadoret and Cain 1981; Cadoret 1986) and aggressivity (Cadoret et al. 1995). In the latter study, however, the genetic predisposition inherited from a birth parent with antisocial personality disorder increased conduct disorder and aggressivity only in adoptees raised in an environment with additional adverse factors (e.g., an adoptive parent suffering from a psychiatric or marital problem) (figure 3) (Cadoret et al. 1995).

Findings from the study of this type of gene-environment interaction may suggest points of intervention, thereby helping to prevent behavior leading to alcoholism. For instance, in the above example, modifications of the environment (e.g., treatment of the adoptive parents’ problems) could affect the adoptee’s outcome even in the presence of a genetic predisposition.

## Factors Influencing Study Quality

### Obtaining Valid Information

Valid information about the birth parents, the adoptive parents, and the rearing environment is crucial when using adoption studies to assess the influences of genetic and environmental factors on behavior. This information must address the four important sources of influences on the adoptee: the genetic and environmental factors from the birth parents, the parental influences from the adoptive parents, and the adoptive family environment. Thus, a major technical difficulty in adoption studies is arranging for data collection from a wide range of sources, some of which are protected by confidentiality.

Information about the birth parents and their behaviors is necessary to determine which adoptee characteristics may represent phenotypes of a genetic predisposition inherited from the parents (e.g., genes predisposing the adoptee to develop alcoholism). This information can be obtained from the records of the adoption agency, hospitals, social services, and similar sources. In studies of adoptees born out of wedlock, reliable information about birth fathers frequently is lacking. However, recent laws requiring written permission from biological fathers to release children for adoption may improve information collection. For example, if a birth father’s name is available, archival information from hospitalizations, incarcerations, or other records (e.g., death certificates) can be obtained provided that the confidentiality required for such records can be maintained.

Adoption agencies usually can provide information about pregnancy and delivery (i.e., influences of the birth-parent environment). Similarly, agency records can supply a large amount of personal information about the adoptive parents and the rearing environment. This information is especially of interest because adoption studies can measure the influences of specific environmental effects as effectively as the influences of genetic effects. Information about the adoptees themselves also is readily available in most cases.

Ideally, adoption studies would include information obtained by personal interviews with all the people who primarily affect the adoptee’s outcome (i.e., the birth parents, the adoptive parents, the adoptee, and friends of the adoptee). Data collected solely from institutional records, however, such as those from the central registries in Scandinavian countries, also can provide valuable information and, at the very least, be used to identify subjects for direct study. Long-term followup of the adoptees, their birth parents, and their adoptive families would result in even more valid information about behaviors that tend to change over time, such as conduct disorders, alcohol abuse, or depression. Such longitudinal studies could considerably increase the identification of psychopathological behaviors that might go undetected in a study relying only on information gathered during one time period.

### Proband Recruitment

How the probands are recruited also can affect the quality of a study’s conclusions. One potential source of bias is the influence of environmental factors on the selection of proband adoptees in the adoptees’ family method. For example, psychological or social problems in an adoptive family may contribute to the adoptee’s psychopathology. Simultaneously, these problems may prompt the family and the adoptee to seek more treatment and thus increase their chances of being included in a sample of adoptees recruited from a clinic population. Factors such as these may compromise the representativeness of the sample.

Similarly, refusal rates among potential study participants could influence the quality of the data obtained. For example, it is possible that adoptees and their families who refuse to participate in a study as a group are distinguished by certain qualities (e.g., personality characteristics). Consequently, their refusal could reduce the representativeness of the study sample.

## Generalizability of Adoption Studies

Whether the findings from adoption studies can be used to draw general conclusions about the contribution of both genetic and environmental factors to the development of alcoholism depends largely on how representative the adoptee sample is. Representativeness, in turn, is determined by variables, such as the criteria for proband selection. Although many of these variables can be controlled for or at least recognized, the inherent biases in adoption practices (e.g., selective placement and predominant recruitment of adoptive families from certain population groups) limit generalizability.

## Summary

Despite the existing limitations and the technical problems associated with conducting adoption studies, the adoption paradigm provides important information about the significance of specific genetic and environmental factors in human behavior. In addition, adoption studies allow researchers to identify specific genetic-environmental interactions that could be relevant for designing early interventions for behaviors that predispose a person to alcohol abuse and dependence.

## Footnotes

1 For a definition of this and other technical terms used in this article, see central glossary, pp. 182–183.

2 An odds ratio is a measure of association between two variables.
